# Essential Indicators Identifying Chronic Inorganic Mercury Intoxication: Pooled Analysis across Multiple Cross-Sectional Studies

**DOI:** 10.1371/journal.pone.0160323

**Published:** 2016-08-30

**Authors:** Stefan Doering, Stephan Bose-O’Reilly, Ursula Berger

**Affiliations:** 1 Institute and Outpatient Clinic for Occupational, Social and Environmental Medicine, WHO Collaborating Centre for Occupational Health, University Hospital Munich, Munich, Germany; 2 Department of Medical Information Sciences, Biometrics, and Epidemiology IBE, Ludwig-Maximilians-University Munich, Munich, Germany; 3 Institute of Public Health, Medical Decision Making and Health Technology Assessment, Department of Public Health, Health Services Research and Health Technology Assessment, UMIT (University for Health Sciences, Medical Informatics and Technology), Hall i.T., Innsbruck, Austria; Institute for Health & the Environment, UNITED STATES

## Abstract

**Background:**

The continuous exposure to inorganic mercury vapour in artisanal small-scale gold mining (ASGM) areas leads to chronic health problems. It is therefore essential to have a quick, but reliable risk assessing tool to diagnose chronic inorganic mercury intoxication. This study re-evaluates the state-of-the-art toolkit to diagnose chronic inorganic mercury intoxication by analysing data from multiple pooled cross-sectional studies. The primary research question aims to reduce the currently used set of indicators without affecting essentially the capability to diagnose chronic inorganic mercury intoxication. In addition, a sensitivity analysis is performed on established biomonitoring exposure limits for mercury in blood, hair, urine and urine adjusted by creatinine, where the biomonitoring exposure limits are compared to thresholds most associated with chronic inorganic mercury intoxication in artisanal small-scale gold mining.

**Methods:**

Health data from miners and community members in Indonesia, Tanzania and Zimbabwe were obtained as part of the Global Mercury Project and pooled into one dataset together with their biomarkers mercury in urine, blood and hair. The individual prognostic impact of the indicators on the diagnosis of mercury intoxication is quantified using logistic regression models. The selection is performed by a stepwise forward/backward selection. Different models are compared based on the Bayesian information criterion (BIC) and Cohen`s kappa is used to evaluate the level of agreement between the diagnosis of mercury intoxication based on the currently used set of indicators and the result based on our reduced set of indicators. The sensitivity analysis of biomarker exposure limits of mercury is based on a sequence of chi square tests.

**Results:**

The variable selection in logistic regression reduced the number of medical indicators from thirteen to ten in addition to the biomarkers. The estimated level of agreement using ten of thirteen medical indicators and all four biomarkers to diagnose chronic inorganic mercury intoxication yields a Cohen`s Kappa of 0.87. While in an additional stepwise selection the biomarker blood was not selected, the level of agreement based on ten medical indicators and only the three biomarkers urine, urine/creatinine and hair reduced Cohen`s Kappa to 0.46. The optimal cut-point for the biomarkers blood, hair, urine and urine/creatinine were estimated at 11. 6 μg/l, 3.84 μg/g, 24.4 μg/l and 4.26 μg/g, respectively.

**Conclusion:**

The results show that a reduction down to only ten indicators still allows a reliable diagnosis of chronic inorganic mercury intoxication. This reduction of indicators will simplify health assessments in artisanal small-scale gold mining areas.

## Introduction

Mercury (Hg) is used to extract gold from ore, especially in artisanal small-scale gold mining (ASGM). Miners working in ASGM are particularly exposed to mercury vapours when they smelt a mercury containing compound, the so called amalgam. To a lesser extent miners are exposed whilst mixing liquid mercury into the grinded, gold containing ores. As a consequence mining villages are highly contaminated with mercury leading to a considerable mercury exposure of the general population [1–3]. The continuous exposure to inorganic mercury vapour in ASGM areas leads to chronic health problems, as published in several papers and reviews [4–30]. These articles focus on different aspects; many of them report human biomonitoring results, indicating the high exposure of the miners and the general population; fewer articles concentrate on clinical symptoms related to chronic inorganic mercury exposure. Mercury is a known neurotoxin, damaging especially the cerebellum [31–33]. As to be expected exposed villagers and miners show a higher prevalence of neurological symptoms [6, 15–17]. The work group “Paediatric Environmental Epidemiology” at LMU together with many international partners had published some of these papers reporting about field projects in Indonesia, Mongolia, Philippines, Tanzania and Zimbabwe [5, 6, 15–17, 34–36]. These papers were based on extensive scientific health assessments, using the analysis of mercury in several specimens, extensive health questionnaires, medical and neuro-psychological examinations. ASGM is performed in approximately fifty to seventy countries globally and up to fifty to one hundred million people are exposed in mining villages. Drasch and Bose-O’Reilly published provides an extensive toolkit to assess the health situation of ASGM miners and their family members [16]. However, so far there is no practical toolkit to enable conducting a rapid assessment. Moreover, it is difficult in different countries to analyse mercury in diverse specimens, due to lack of laboratory capacities. The aim of this work is to propose a low-cost, easy applicable and robust toolkit with a reduced set of indicators to allow for a fast identification of chronic inorganic mercury intoxication. We re-evaluated the existing data sets from Indonesia, Philippines, Tanzania and Zimbabwe to reduce the set of indicators for CIMI to the most essential ones. In addition, we assess the impact of different biomarkers on the goodness of the toolkit, to explore, whether all biomarkers are needed for a reliable diagnosis of CIMI. A simple toolkit for a fast identification of CIMI is of paramount importance for the risk assessment in mercury hot spots and for an assessment of the prevalence in ASGM areas.

## Materials and Methods

For this publication data from several epidemiological cross sectional studies in different artisanal small-scale gold mining communities were used (see Table 1) [6, 15–17, 29, 37–44]. All participants were volunteers and had signed a written consent form. United Nations Industrial Development Organization (UNIDO), United Nations Development Programme, (UNDP), World Health Organization (WHO) and Ludwig-Maximilians University Munich (LMU) had fulfilled the country specific demands and regulations to perform the health assessments. All studies obeyed the relevant national, state, and local regulations, the appropriate regional health authorities and the national ministries of health had given all necessary permission, including extensive legal, formal, and ethical considerations.

**Table 1:** Data sources for the analysis and imputation process.

Country, area	Year	Study design	Project partners	References
Indonesia	2003	Miners, males and females, children; mining community from Galangan (Kalimantan) and Takawaan (Sulawesi); control group from Air Mandidi (Sulawesi)	CETEM, LMU, UNIDO	[6, 10, 42]
Mongolia	2008	Women at child bearing age; mining communities Bornuur sum, Jargalant sum; control group from Khushaat sum	MoH, LMU, UMIT, WHO	[17, 34]
Philippines	1999	Miners, males and females, children; mining community from Mt. Diwata and Monkayo; control group from Davao	BGS, LMU, UNIDO	[16, 38, 39]
Tanzania	2003	Miners, males and females, children; mining community from Rwamagasa; control group from Katoro	BGS, LMU, UNIDO	[15, 37, 42]
Zimbabwe	2004	Miners, males and females, children; mining community Kadoma; control group from Chikwaka	BRGM, LMU, UNIDO	[29, 35, 41, 43]
Zimbabwe	2006	Women at child bearing age and their breast fed infants; mining community Kadoma; control group from Chikwaka	LMU, UoZ	[29, 43]

### Data sources

Data from the various projects were pooled together in a data base and re-analysed [5]. The different ASGM areas were in Indonesia, Philippines, Tanzania and Zimbabwe [6, 15–17]. Participants included in the analyses are aged from 7 to 64 years. Young children and elderly people were excluded to avoid age specific effects [36]. 25 participants with risk factors that can mimic neurological symptoms (acute alcohol consumption) or pre-existing neurological diseases (stroke and Parkinson’s disease) were excluded from further analyses. In addition, 97 participants with an unclear exposure history or participants who moved from exposed to non-exposed areas or vice versa were excluded. The study protocols differ between the different countries, especially with respect to the number of obtained indicators and characteristics. The studies from Indonesia, Tanzania and Zimbabwe 2004 contain all relevant indictors and were therefore used for the main analyses resulting in a sample of 884 participants. Basic information on this study population can be found in Table 2. The different studies were performed as health assessments in different countries. The study in the Philippines 1999 was the first assessment and the study protocol was less extensive. The assessments in Indonesia, Tanzania and Zimbabwe in 2003/2004 were the most complete studies and have been performed in exactly the same way. The studies in Mongolia 2008 and Zimbabwe 2006 focused on women at child bearing age and the study protocol was more restricted due to financial limitations. For the imputation process for missing values all studies were used to improve results. For the final analysis only the three assessments from Indonesia, Tanzania and Zimbabwe 2003/2004 we used (n = 618).

**Table 2:** Basic information about participants.

**Number of participants used for the main analysis (by country)**	**Non exposed group**	**Low exposed group**	**Medium exposed group**	**High exposed group**	**Total number**
Indonesia	21	188	94	144	427
Tanzania 2003	31	54	35	104	224
Zimbabwe 2004	48	33	20	132	233
**Total**	**100**	**255**	**149**	**380**	**884**
**Number of participants used for the missing value imputation (by country)**	**Non exposed group**	**Low exposed group**	**Medium exposed group**	**High exposed group**	**Total number**
Zimbabwe 2006	43	54	13	13	123
Mongolia	42	92	13	50	197
Philippines 1999	41	163	54	40	298
**Total**	**126**	**309**	**80**	**103**	**618**
**Total over all countries**	**226**	**564**	**229**	**483**	**1502**
					
**Main characteristics of participants included in the main analysis, by group of exposure**					
**Variable**	**Non exposed group**	**Low exposed group**	**Medium exposed group**	**High exposed group**	**Total number**
age in years (median)	27.61 (27)	24.93 (23)	25.26 (22)	30.46 (30)	27.66 (27)
female / male	47 / 53	181 / 74	75 / 74	69 / 311	372 / 512
male in % of total	53.0%	29.0%	49.7%	81.8%	57.9%

### Defining exposure

According to the different levels of exposure the data were stratified:

Non exposed group: control groups, with no specific contact to mercury, but comparable social background as exposed groups (n = 100)

Low exposed group: participants living in an exposed area, but no mining activities related to mercury (n = 255)

Medium exposed group: participants working with mercury as a panner (n = 149)

High exposed group: participants working with mercury as an amalgam smelterer (n = 380)

### Health assessment

Every participant was interviewed by a nurse using consistent, translated questionnaires. Information was collected on: (a) Exposure situation including work exposure, duration of exposure, contact to mercury at home. (b) confounders such as age, gender, alcohol consumption, pesticide exposure and fish consumption, (c) conflicting health problems such as neurological diseases, accidents and infectious diseases(d) health problems known to be possibly related to CIMI (excessive salivation, metallic taste, sleeping problems, headaches and memory problems, loss of hair). In addition, every participant was medically examined. Clinical and anamnesis data such as grey to bluish discoloration of the oral cavity, hypomimia, number of amalgam fillings were obtained. Since mercury is a neurotoxin, a strong focus was given to neurological signs and symptoms such as ataxia, coordination problems, tremor and abnormal reflexes, based on the textbook “Scales and scores in neurology” [45]. Some simple neuropsychological tests were used to quantify tremor, ataxia, coordination and memory problems:

- Digit span test to test the short term memory (part of Wechsler Memory Scale) [45]
- Matchbox test to test for coordination, intentional tremor and concentration [46]
- Frostig Score (subtest Ia 1–9) to test for tremor and visual-motoric capacities [47]
- Pencil tapping test to test for intentional tremor and coordination [45]

In the supplement a detailed description of the assessment methods and neuropsychological test is given (S1, S2 and S3 Tables). As proteinuria is a typical sign of kidney damage due to mercury, a commercial kit (Bayer^®^) was used to test for proteinuria.

### Mercury analysis in urine, blood and hair

Human specimens were collected from the participants, containing urine, blood and hair. The Institute of Forensic Medicine (LMU) performed most analyses using a Cold-Vapor Atomic-Absorption-Spectrometer (Perkin-Elmer 1100 B spectrometer). Urine and hair of the non-exposed group in Mongolia were analysed by the National Institute for Minamata Disease (Minamata, Kumamoto, Japan) with a Cold-Vapor Atomic-Absorption-Spectrometer (CV-AAS) [17]. Blood of the non-exposed group in Mongolia was analysed by the Health and Safety Laboratory (Harpur Hill, Buxton, United Kingdom) with an Inductively-Coupled-Plasma Mass-Spectrometer (ICP-MS). Inorganic mercury was analysed by LMU using Lumex mercury Cold-Vapor Atomic-Absorption-Spectrometer (RA915+, Lumex Ltd^®^, St. Petersburg, Russia). Urinary mercury levels were adjusted for creatinine to correct for urine excretion rates [48]. Sufficient stringent external and internal quality control was assured. More detailed information about the methods to analyse mercury in the different specimens and quality control questions can be found in the supplement and different previously published papers.

### Risk assessment

The data from the first project in the Philippines had been used to develop an algorithm to diagnose CIMI [16]. The algorithm combines a set of anamnestic or clinical indicators and symptoms plus neuropsychological parameters and proteinuria to a medical score sum (see Table 3). To avoid an observer bias the clinical and neuropsychological examinations were performed by the same person in each project region. The higher the score the more likely is the intoxication.

**Table 3:** Parameters of the medical sum score for chronic inorganic mercury intoxication.

Test	Established medical score sum^(a)^	Revised medical score sum
Anamnestic data *		
Metallic taste	0/1	-
Excessive salivation	0/1	0/1
Tremor at work	0/1	0/1
Sleeping problems at night	0/1	0/1
Health problems worsened since Hg exposed	0/1	-
Clinical data *		
grey to bluish discoloration of the oral cavity	0/1	0/1
Ataxia of gait	0/1	0/1
Finger to nose tremor	0/1	-
Dysdiadochokinesia	0/1	0/1
Heel to shin ataxia	0/1	0/1
Proteinuria	0/1	0/1
Neuropsychological tests **		
Matchbox test	0/1	0/1
Pencil tapping test	0/1	0/1
**Maximum sum score**	**13**	**10**

Coding of the parameters of the medical score sum

* Anamnestic and clinical data: 0 = no symptom, 1 = pathological symptom

** Neuropsychological tests: 0 = first quartile, 1 = worse performance than first quartile.

(a) Medical sum score as proposed by Drasch et al [16]

The German Human Biomonitoring Commission has established reliable exposure limit values for several substances [5, 16, 49]. For hair, similar levels of mercury were used as proposed by Drasch et al [16]. Exposure limit values used in the score to identify CIMI can be found in Table 4 [16, 49, 50].

**Table 4:** Exposure limit values of mercury in urine, urine/creatinine, blood and hair.

	Hg-urine (μg/l) ^1^	Hg-urine/crea.(μg/g crea.) ^1^	Hg-blood (μg/l) ^1^	Hg-hair (μg/g) ^2^	
**Below 1**^**st**^ **exposure limit value**	**≤ 7**	**≤ 5**	**≤ 5**	**≤ 1**	**Low level**
**Between 1st to 2**^**nd**^ **exposure limit value**	**> 7 to ≤ 25**	**> 5 to ≤ 20**	**> 5 to ≤ 15**	**> 1 to ≤ 5**	**Alert level**
**Over 2**^**nd**^ **exposure limit value**	**> 25**	**> 20**	**> 15**	**> 5**	**High level**

^1^ HBM = Human Biomonitoring values [49]

^2^ Exposure limits of Methyl mercury in hair [16, 50]

The subsequent categories were used:

Category 0: Below 1^st^ exposure limit
Category 1: From 1^st^ to 2^nd^ exposure limit
Category 2: Over 2^nd^ exposure limit

Increased levels of mercury in the specimen together with typical signs and symptoms were finally combined in an algorithm to decide if a participant was considered as intoxicated. Hence, the medical diagnosis of mercury intoxication is based on the medical score and on increased mercury levels in urine, blood and hair [16] (see Table 5).

**Table 5:** Algorithm for risk assessment of chronic inorganic mercury intoxication [16].

		Medical Score Sum		
		0–2	3–4	5–10
**Hg in all specimens**	**< 1**^**st**^ **exposure limit value**	–	**–**	**–**
**Hg at least in one specimen**	**> 1**^**st**^ **exposure limit value**	**–**	**–**	+
	**> 2**^**nd**^ **exposure limit value**	**–**	+	+

Decision:– = no intoxication, + = intoxication; HBM = Human Biomonitoring value

## Statistical Analysis

### Multiple Imputation for missing values

To avoid big loss of data due to missing values a non-parametric multiple imputation (MI) of missing values was performed, using the R-package MICE combined with the R-package missForest [51]. To improve the MI results we performed imputation in two steps. In the first step a pooled dataset was used including the data from three main studies of Indonesia (2003), Tanzania (2003) and Zimbabwe (2004) as well as additional data from studies in Mongolia, Zimbabwe (2006) and the Philippines (1999) (see Table 2) and missing data in those indicators, which were available for all six studies, were imputed. In a second step we imputed data for the remaining indicators based on the data of the three main studies Mongolia, Zimbabwe (2006) and the Philippines (1999). Five imputed datasets were constructed. Results of the analyses, i.e. regression coefficients and descriptive statistics, were pooled according to Rubin`s rule [52] using the R-package Zelig. A sensitivity analysis comparing results of imputed and unaltered data was done to assess the quality of the MI.

### Sensitivity analysis of biomarker exposure limits

A sensitivity analysis was performed comparing established exposure limit values of the biomarkers mercury in hair, blood, urine and urine/creatinine to the optimal cut-points found in the data. This outcome-based optimal cut-point search determines the cut-point, which discriminates best with respect to the outcome of CIMI using chi-square tests [53]. A Bonferroni-correction was performed to avoid inflation of the Type I error. The unadjusted alpha was set to 5%. Every p-value was plotted against its cut-point on a–log scale.

#### Variable selection in logistic regression

A stepwise variable selection in logistic regression was performed to identify the most essential indicators for a diagnosis of CIMI. Three nested scenarios were defined:

**Scenario 1:** model selected from all measurements and characteristics (including all four biomarkers: mercury concentration in urine, in urine/creatinine, in blood and in hair)

**Scenario 2:** model selected from all measurements and characteristics, excluding the biomarker mercury concentration in blood

**Scenario 3:** model selected from all measurements and characteristics, excluding the two biomarkers mercury concentration in blood and in hair

A complete list of all measurements and characteristics can be found in S1, S2 and S3 Tables.

Variable selection and comparison of goodness of fit of the three models is based on the Bayesian information criterion (BIC) following the publication of Wood et al [54].

Indicators selected in scenario 1 were used to define the new, reduced medical sum score to diagnose CIMI.

#### Optimal cut-points for the reduced medical sum score and the level of agreement

The level of agreement (Cohen`s Kappa) was used to determine the optimal cut-points for the reduced medical sum score, which identifies the intoxicated patients [55]. A Cohen`s Kappa of 1 indicates perfect agreement. According to these cut-points the medical score sum was categorized into three groups. To validate the resulting diagnostic procedure the level of agreement (Cohen`s Kappa) with the established diagnostic procedure was assessed and sensitivity and specificity were compared. All analyses have been performed using R Statistical Software version 3.0.3 and the R-packages Zelig, missForest, MICE and ggplot2.

## Results

The analysis contained overall 884 participants. Hereby, 427 (48.3%) were from Indonesia, 224 (25.3%) from Tanzania and 233 (26.4%) from Zimbabwe. The average age was 27.6 years with 372 (42.1%) female and 512 (57.9%) male.

### Multiple Imputation

From 884 participants in the data, 325 (36.8%) persons had incomplete information in at least one of the variables of interest. The descriptive statistics for complete cases and imputation data showed similar results. A sensitivity analysis on the final models comparing the results of a complete case analysis and the analysis based on imputation data showed consistently overlapping 95% confidence intervals and did not indicate any violation of the assumption of data missing at random (MAR). We therefore based all analyses on the five imputed data sets. A comprehensive overview is given in S1, S2 and S3 Tables).

### Pre-selection of indicators

The original algorithm, developed by Drasch et al [16], included 17 anamnestic, clinical and neuropsychological parameters. They had been selected since these 17 parameters showed a statistical significance versus the exposure situation. During the following three UNIDO projects in Indonesia, Tanzania and Zimbabwe 2003 and 2004 this algorithm was used once again. Univariate analysis of data from these three projects displayed, that the mento-labial reflex, memory test and Frostig test had no statistical significant association with the outcome [6, 15, 36, 37, 42].

### Sensitivity analysis of biomarker threshold limits

**Fig 1:**
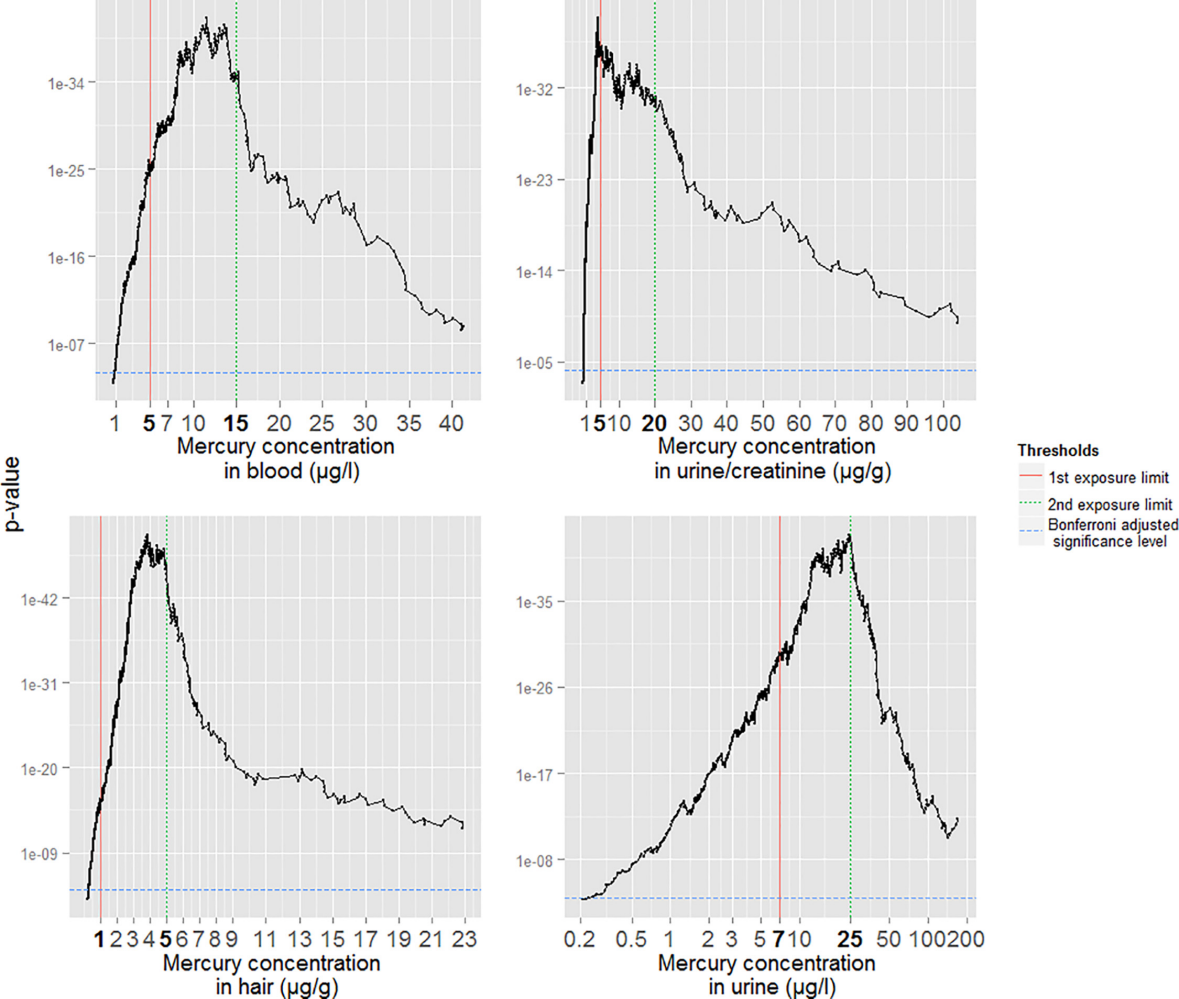
Sensitivity analysis of biomarker threshold limits.

Cut-points and exposure limit values with associated p-values for the mercury concentration in blood, hair, urine and urine /creatinine are depicted in Fig 1.

The association of both exposure limit values of the biomarker blood was highly significant with a p-value of 4 x e^-25^at 5 μg/l and 2 x e^-35^at 15 μg/l. The optimal cut-point was estimated at 11.6 μg/l with a p-value of 1 x e^-45^. The Bonferroni adjusted significance level is 0.0001. The biomarker of mercury in hair had highly significant p-values of 2 x e^-15^ at 1 μg/g and 7 x e^-46^ at 5 μg/g for the exposure limit values. The optimal cut-point was estimated at 3.8 μg/g with a p-value of 1 x e^-52^. The Bonferroni adjusted significance level is 6 x e^-05^. The biomarker of mercury in urine had highly significant p-values at the exposure limit values with values of 5 x e^-30^ at 7 μg/l and 2 x e^-42^at 25 μg/l. The optimal cut-point was estimated at 24 μg/l with a p-value of 8 x e^-42^. The Bonferroni adjusted significance level is 8 x e^-05^. For the biomarker of mercury in urine/creatinine the p-values of 1 x e^-36^ at 5 μg/g and 2 x e-^31^ at 20 μg/g were also highly significant The optimal cut-point was estimated at 4 μg/g with a p-value of 3 x e^-39^. The Bonferroni adjusted significance level is 6 x e^-05^.

### Selection of relevant indicators in logistic regression

Variable selection applied to scenario 1 and 2 resulted in the same model with an BIC of 349.77. Ten of the 13 indicators used to calculate the medical sum score were selected in the model. From the anamnestic indicators three of five, from clinical indicators five of six and both neuropsychological indicators were selected. From the medical score the indicators, metallic taste, health problems worsened since mercury exposure and finger-to-nose tremor were not selected. From the biomarkers, mercury concentration in hair and mercury concentration in urine were selected. No other additional indicator or confounders were selected in the stepwise selection algorithm. Scenario 3 had an averaged BIC of 463.06. Nine of thirteen variables used to build the medical sum score were selected. Metallic taste, tremor at work, health problems worsened since mercury exposure and finger-to-nose tremor were not selected. From the biomarkers, mercury concentration in urine and mercury concentration in urine/creatinine were selected. In addition, the indication loss of hair was selected. An overview of the variable selection is provided in Table 6 and in more detail in S2 and S3 Tables.

**Table 6:** Overview of variable selection models.

	Model1 ^1^	Model 2 ^2^	Model 3 ^3^
Bayesian Information Criterion ^4^	349.77	349.77	463.06
***Medical Indicators used in risk assessment***			
*Anamnestic variables*			
Excessive salivation	X	X	X
Tremor at work	X	X	
Sleeping problems at night	X	X	X
*Clinical variables*			
Grey to bluish discoloration of the oral cavity	X	X	X
Heel to shin ataxia	X	X	X
Dysdiadochokinesis	X	X	X
Proteinuria	X	X	X
Ataxia of gait (walking)	X	X	X
*Neuropsychological tests*			
Pencil tapping-test	X	X	X
Matchbox- test	X	X	X
***Biomarkers used in risk assessment***			
Hg in blood (μg/l)		omitted *	omitted *
Hg in hair (μg/g)	X	X	omitted *
Hg in urine (μg/l)	X	X	X
Hg urine/creatinine (μg/g)			X
***Additional indicators and confounders with sign*. *effects not used in risk assessment***			
Loss of hair			X

^1^ Full model with all covariates (mercury in urine, urine/creatinine, blood and hair as biomarkers)

^2^ Full model with all covariates, except variable mercury concentration in blood

^3^ Full model with all covariates, except variables mercury concentration in blood and hair

^4^ Averaged over five imputation datasets

* Not included in this model selection algorithm

### Level of agreement between established and revised inorganic mercury intoxication score

A new medical sum score was defined according to the result of the variable selection. This medical sum score consists of ten instead of thirteen indicators (see Table 3). The anamnestic data consists of excessive salivation, tremor at work and sleep problems at night. Clinical data consists of bluish coloration of gingiva, ataxia of gait, dysdiadochokinesia, heel to shin ataxia and proteinuria. The neuropsychological tests include both the matchbox-test and the pencil tapping test. Comparison of the revised medical sum score of CIMI with biomarker blood and the identification of mercury intoxication based on the established score yields in an optimal Cohen’s Kappa of ĸ = 0.92 at a score of three and of six. Sensitivity and specificity are 100.0% and 96.6%, respectively. The revised medical sum score without the biomarker blood has a maximal Cohen`s Kappa as low as 0.49 at a score of three and of five, with a sensitivity and specificity of 94.5% and 85.0%. A comparison between established and revised risk assessment of CIMI by exposure group is provided in Table 7.

**Table 7:** Shift table, sensitivity and specificity of established and the reduced risk assessment score to assess chronic inorganic mercury intoxication by exposure group^1^.

		Established risk score			
Revised risk score		Yes	No	Sensitivity	Specificity
Control	Yes	0	0	Not defined	1
	No	0	70		
Low exposure	Yes	22	2	1	0.991
	No	0	213		
Medium exposure	Yes	28	2	1	0.983
	No	0	114		
High exposure	Yes	104	17	1	0.924
	No	0	206		
**Overall**	**Yes**	**154**	**21**	**1**	**0.964**
	**No**	**0**	**603**		

^1^Based on complete cases omitting all cases with missings

## Discussion

The goal of this work was to develop a practical toolkit to enable conducting a rapid assessment of the health situation of gold miners and their families. This is realized by selecting the most important indicators from an existing assessment toolkit already in use in ASGM gold mining areas. Furthermore, the sensitivity of already established biomarker threshold limits is analysed.

### Sensitivity analysis of biomarker threshold limits

#### Mercury in blood

The second exposure limit is close to the determined optimal cut-point. The associated p-value is comparable to the p-value of the optimal cut-point. The p-value of the first exposure limit is statistically highly significant, but the data does not support the importance of the second exposure limit for the diagnosis of CIMI.

#### Mercury in hair

The range between 3.5 μg/g and 5 μg/g contains the smallest p-values of comparable magnitude. This range includes the determined optimal cut-point and second exposure limit. Hence, the cut-point search supports the validity of the second exposure limit. However, there is no evidence to support the validity of the first exposure limit at 1 μg/g. Both exposure limits were derived from the exposure to methyl-mercury which has a different toxicokinetic profile compared to inorganic-mercury as in our research question [56].

#### Mercury in urine

The second exposure limit and the determined optimal cut-point are almost identical. The first exposure limit is statistically highly significant, but the importance of this particular cut-point is not supported by the data.

#### Mercury in urine/creatinine

1^st^ and 2^nd^ exposure limit are supported by our data. The 1^st^ exposure limit is also close to the determined optimal cut-point.

### Threshold levels

The optimal cut-point search for the biomarkers blood, urine, urine/creatinine and hair did not always confirm the exposure limit values. The association between the exposure limits and outcome is always highly significant. However the strength of the association is often comparably low. The close range around the given exposure limit did not include the optimal cut-point. However, a sensitivity analysis is not sufficient to make statistical inference and was also not the primary goal of this study. Missing confidence intervals make conclusions difficult and the optimal cut-point estimates might overfit the data.

### Selection of relevant indicators

In three scenarios we have investigated which of the indicators proposed in the original medical score add relevant information when diagnosing CIMI, and examined how robust a diagnosis is when certain biomarkers are not available. The purpose of the first scenario is to identify all relevant indicators including all biomarkers from hair, urine, urine/creatinine and blood. Most of the indicators of the original medical sum score have been verified by the variable selection: Anamnestic data, clinical data as well as neurophysiological tests seem to be important for the diagnosis. However anamnestic data are proportionally less often selected compared to the other groups of variables. However, not all anamnestic indicators have been selected. This seems plausible because these indicators rely on self-assessment and memory and are therefore more likely to be flawed. From the six clinical indicators, all but finger to nose tremor add relevant information to the diagnosis of CIMI. All together we could reduce the number of relevant indicators from thirteen to ten. For the additional confounders such as demographic characteristics, alcohol and fish consumption, pesticide exposure and related health problems we found no relevant additional contribution to the diagnosis. Concerning the impact of biomarkers this scenario already reveals that the mercury concentration in blood does not improve the goodness of fit when modelling diagnosis of CIMI taking all the other indicators and biomarkers into account and thus is not selected by the stepwise variable selection algorithm. Hence, when omitting the biomarker blood in the second scenario, the resulting model does not change. The third scenario showed that a further reduction of the score by the biomarker mercury concentration in hair results in a considerable loss in the goodness of the medical score. It was replaced in this scenario by the information on hair loss. Therefore it can be concluded that the biomarker mercury concentration in hair is an essential indicator for the diagnosis of CIMI.

### Level of agreement between revised and old inorganic mercury intoxication variable

The results for the revised medical score diagnosing CIMI including the biomarkers urine, urine/creatinine, hair and blood show a high level of agreement with the established medical sum score, and also sensitivity and specificity are high. Thus, the reduction from thirteen to ten indicators still allows a reliable diagnosis of CIMI. However, an additional reduction of the score by the biomarker mercury concentration in blood reduces the level of agreement notably, as well as it results in a lower sensitivity and specificity. This means that the biomarker blood remains an important indicator for a reliable diagnosis of CIMI. Even after the reduction of variables it is necessary to analyse mercury in human specimens, which is not so easily possible in many countries where ASGM is common. Laboratory capacities need to be increased to be able to perform mercury analysis, not only to diagnose a CIMI, but as well to monitor mercury levels in the population, e.g. before and after technical interventions to reduce the mercury exposure for the population. There are mobile mercury analysers which can be used in remote areas and which are not too expensive [57].

### Limitations

The result of the selection of relevant indicators showed that the biomarker blood did not meet the statistical selection criterion. Certainly, this is due to the fact, that biomarkers were correlated and thus carried partially same information with respect to diagnosing CIMI. However the significantly different levels of agreement comparing the risk assessment with and without blood could not confirm the unimportance of mercury in blood as an indicator. The reason for this is that we chose a rather conservative criterion in the stepwise selection, to achieve a reduction of the numbers of indicators incorporated in the diagnosis score. There is no gold standard to diagnose CIMI. In our opinion the best available tool so far was the algorithm of Drasch et al [16]. This surrogate outcome, as well as our modified surrogate outcome is a predictor of the latent disease state and therefore biased to an unknown extent. This also implies that it is only possible to find a better subset of indicators in terms of parsimony. But this is not true in terms of prediction, as there is no way to validate the results. Furthermore, the association between the outcome and the variables used in risk assessment to determine the outcome variable is likely to be overestimated. This holds true for the variable selection, but also for the optimal cut-point search where the exposure limits also were used to define the outcome variable. Confidence intervals were not estimated from the optimal cut-points. Statistical inferences are therefore not possible. Exposure limit of mercury in blood, urine and urine/creatinine are accepted threshold values for the common population in high income countries derived from a mixed exposure with methyl-mercury and inorganic exposure. The upper reference values for mercury in hair were taken from the publication of van Wijngaarden et al [50]. The lower reference value was defined by the US Environmental Protection Agency, both for the exposure to methyl-mercury. The toxicokinetic profiles of methyl-mercury and inorganic mercury are different. However, it was also shown that the proportion of inorganic mercury increases with higher values of total mercury in hair. This unavoidable restriction of available exposure limits (no specific exposure limits for inorganic mercury and no exposure limits for the purpose ASGM) might have biased our results. The data of our study comes from cross-sectional surveys, and the causative mercury exposures are unknown.

## Conclusion

The results show that a reduction down to only ten indicators still allows a reliable diagnosis of CIMI. The level of agreement decreased considerably when excluding the biomarkers blood. This reduction of indicators will simplify health assessments in ASGM areas. Scientists and health care providers are encouraged to use these ten essential indicators to identify miners and other mercury exposed people with chronic inorganic mercury intoxication. The exposure limits values being used could partly not be confirmed. Therefore further research is needed to investigate if exposure limits for inorganic mercury in ASGM need to be adjusted. It might be useful to pool biomonitoring data from different ASGM projects and to apply more advanced statistical methods. This could lead to define improved exposure limit values for mercury in ASGM areas.

## Supporting Information

S1 Table Pre-and post-imputation descriptives of all study variables. (PDF)

S2 Table Model 1 and 2 after stepwise variable selection in logistic regression. (PDF)

S3 Table Model 3 after stepwise variable selection in logistic regression. (PDF)
