# Supplementary material for: Essential Indicators Identifying Chronic Inorganic Mercury Intoxication: Pooled Analysis across Multiple Cross-Sectional Studies
Source: PLoS One. 2016 Aug 30;11(8):e0160323. doi: 10.1371/journal.pone.0160323 (PMC5004870; doi:10.1371/journal.pone.0160323)
Supplement: S1 Table — (PDF) [file pone.0160323.s001.pdf]

Additional file 1: Pre-and post-imputation descriptives of all study variables

Variable	Pre-imputation		Post imputation (N=884)		NA (%)
	Mean (SD)	N (%)	Mean (SD)	%	
**Gender**					0.0 %
Male		512 (57.9 %)		57.9 %	
Female		372 (42.1 %)		42.1 %	
**Hg in urine (µg/l)**	42.75 (216.29)		42.83 (215,90)		0.3 %
**Hg in blood (µg/l)**	12.56 (28.67)		12.70 (28.75)		0.6 %
**Hg in hair (µg/g)**	7.39 (35.18)		7.66 (34.1)		8.5 %
**Hg urine/crea (µg/g)**	22.68 (82.45)		22.64 (82.25)		0.6 %
**Exposure group**					0.0 %
Control		100 (11.3 %)		11.3 %	
low exposed		255 (28.8 %)		28.8 %	
medium exposed		149 (16.9 %)		16.9 %	
high exposed		380 (43.0 %)		43.0 %	
**Metallic taste**					0.0 %
No		788 (89.1 %)		89.1 %	
Yes		96 (10.9 %)		10.9 %	
**Excessive salivation**					0.0 %
No		751 (85.0 %)		85.0 %	
Yes		133 (15.0 %)		15.0 %	
**Sleep disturbances**					0.0 %
No		690 (78.1 %)		78.1 %	
Yes		194 (21.9 %)		21.9 %	
**Loss of hair**					1.7 %
No		744 (85.6 %)		85.7 %	
Yes		125 (14.4 %)		14.3 %	
**Tremor at work**					0.1 %
No		759 (86.0 %)		86.0 %	
Yes		124 (14.0 %)		14.0 %	
**Feel nervous**					0.2 %
No		793 (89.9 %)		89.8 %	
Yes		89 (10.1 %)		10.2 %	
**Palpitations**					0.1 %
No		738 (83.6 %)		83.5 %	
Yes		145 (16.4 %)		16.5 %	
**Headache**					0.2 %
No		635 (72.0 %)		72.0 %	
Yes		247 (28.0 %)		28.0 %	
**Nausea**					0.1 %
No		757 (85.7 %)		85.7 %	
Yes		126 (14.3 %)		14.3 %	
**Feel numbness, prickling, aching**					0.0 %
No		681 (77 %)		77 %	
Yes		203 (23 %)		23 %	
**Tired easily**					0.0 %
No		670 (75.8 %)		75.8 %	
Yes		214 (24.2 %)		24.2 %	
**Need to rest more**					0.2 %
No		730 (82.8 %)		82.7 %	
Yes		152 (17.2 %)		17.3 %	
**Feel sleepy or drowsy**					0.1 %
No		745 (84.4 %)		84.3 %	
Yes		138 (15.6 %)		15.7 %	
**No longer able to start anything**					0.5 %
No		845 (96.0 %)		96.0 %	
Yes		35 (4.0 %)		4.0 %	
**Always lack energy**					0.1 %
No		730 (82.7 %)		82.6 %	

Yes		153 (17.3 %)		17.4 %	
**Less strength in muscles**					0.1 %
No		734 (83.1 %)		83.1 %	
Yes		149 (16.9 %)		16.9 %	
**Feel weak**					0.2 %
No		721 (81.7 %)		81.7 %	
Yes		161 (18.3 %)		18.3 %	
**Get weak as go on**					0.0 %
No		747 (84.5 %)		84.5 %	
Yes		137 (15.5 %)		15.5 %	
**Concentrating problems**					0.1 %
No		760 (86.1 %)		86.0 %	
Yes		123 (13.9 %)		14.0 %	
**Problems to think clear**					0.0 %
No		810 (91.6 %)		91.6 %	
Yes		74 (8.4 %)		8.4 %	
**Problems to find correct words**					0.1 %
No		849 (96.1 %)		96.2 %	
Yes		34 (3.9 %)		3.8 %	
**Problems with eyestrain**					0.1 %
No		731 (82.8 %)		82.8 %	
Yes		152 (17.2 %)		17.2 %	
**Memory problems**					0.5 %
No		665 (75.6 %)		75.5 %	
Yes		215 (24.4 %)		24.5 %	
**Teeth with amalgam fillings**					0.0 %
No		883 (99.9 %)		99.9 %	
Yes		1 (0.1 %)		0.1 %	
**Grey to bluish discoloration of the oral cavity**					0.0 %
No		719 (81.3 %)		81.3 %	
Yes		165 (18.7 %)		18.7 %	
**Ataxia of gait (walking)**					0.0 %
No		640 (72.4 %)		72.4 %	
Yes		244 (27.6 %)		27.6 %	
**Finger to nose tremor**					0.0 %
No		827 (93.6 %)		93.6 %	
Yes		57 (6.4 %)		6.4 %	
**Dysdiadochokinesis**					0.0 %
No		627 (70.9 %)		70.9 %	
Yes		257 (29.1 %)		29.1 %	
**Heel to shin ataxia**					0.2 %
No		669 (75.9 %)		75.9 %	
Yes		213 (24.1 %)		24.1 %	
**Heel to shin intentional**					0.2 %
No		872 (98.9 %)		98.8 %	
Yes		10 (1.1 %)		1.2 %	
**Mentolabial reflex**					0.2 %
No		684 (77.6 %)		77.5 %	
Yes		198 (22.4 %)		22.5 %	
**Bradykinesia**					0.3 %
No		779 (88.4 %)		88.4 %	
Yes		102 (11.6 %)		11.6 %	
**Hypomimia**					0.3 %
No		785 (89.1 %)		89.1 %	
Yes		96 (10.9 %)		10.9 %	
**Proteinuria**					0.2 %
No		784 (88.9 %)		88.9 %	
Yes		98 (11.1 %)		11.1 %	
**Matchbox test**	20.81 (5.51)		20.82 (5.5)		0.3 %

**Pencil tapping test**	52.1 (11.99)		52.09 (11.98)		0.7 %
**Age (years)**	27.66 (12.29)		27.66 (12.29)		0.0 %
**Height (cm)**	157.38 (12.9)		157.35 (12.8)		2.5 %
**Weight (kg)**	53.7 (14.8)		53.68 (14.73)		2.5 %
**Years in area (years)**	10.59 (10.54)		10.66 (10.37)		5.2 %
**Storing Hg at home**					1.1 %
Never		311 (35.6 %)		35.7 %	
At work		169 (19.3 %)		19.3 %	
At home		394 (45.1 %)		45.0 %	
**Working cloth at home**					0.2 %
No		270 (30.6 %)		30.6 %	
Yes		612 (69.4 %)		69.4 %	
**Years,working with Hg**	3.12 (4.58)		3.12 (4.58)		0.0 %
**Is a smoker**					0.9 %
No		657 (75 %)		74.8 %	
Yes		219 (25 %)		25.2 %	
**Drinks alcohol**					0.1 %
No		716 (81.1 %)		81.0 %	
Yes		167 (18.9 %)		19.0 %	
**Handling with insecticides**					0.2 %
No		485 (55 %)		55 %	
Yes		397 (45 %)		45 %	
**Medical history of malaria**					0.0 %
No		360 (40.7 %)		40.7 %	
Yes		524 (59.3 %)		59.3 %	
**Fish consumption**					0.8 %
None or low		119 (13.6 %)		13.5 %	
Medium or high		758 (86.4 %)		86.5 %	
**Consumes local chicken**					0.1 %
Never		87 (9.9 %)		9.8 %	
>= once a month		332 (37.6 %)		37.7 %	
>= once a week		370 (41.9 %)		41.8 %	
>= once a day		94 (10.6 %)		10.6 %	
**Consumes local meat**					0.6 %
Never		108 (12.3 %)		12.3 %	
≥ once a month		351 (39.9 %)		39.9 %	
≥ once a week		330 (37.5 %)		37.5 %	
≥ once a day		90 (10.2 %)		10.2 %	
**Consumes local vegetables or fruits**					0.0 %
Never		53 (6.0 %)		6.0 %	
≥ once a month		25 (2.8 %)		2.8 %	
≥ once a week		155 (17.5 %)		17.5 %	
≥ once a day		651 (73.6)		73.6 %	
**Quadrizeps reflex (knee)**					0.1 %
No		720 (81.5 %)		81.5 %	
Yes		163 (18.5 %)		18.5 %	
**Achilles reflex (ankle jerk)**					0.6 %
No		584 (66.4 %)		66.4 %	
Yes		295 (33.6 %)		33.6 %	
**Visual field (radian)**	2.87 (0.24)		2.87 (0.24)		1.9 %
**Health problems**					0.2 %
No		846 (95.9 %)		95.9 %	
Yes		36 (4.1 %)		4.1 %	
