# Supplementary material for: Essential Indicators Identifying Chronic Inorganic Mercury Intoxication: Pooled Analysis across Multiple Cross-Sectional Studies
Source: PLoS One. 2016 Aug 30;11(8):e0160323. doi: 10.1371/journal.pone.0160323 (PMC5004870; doi:10.1371/journal.pone.0160323)
Supplement: S2 Table — (PDF) [file pone.0160323.s002.pdf]

Additional file 2: Model 1 and 2 after stepwise variable selection in logistic regression

Indicator		Pre- imputation	Post- imputation					
		Complete case OR (95% CI)	1st imputation OR (95% CI)	2nd imputation OR (95% CI)	3rd imputation OR (95% CI)	4th imputation OR (95% CI)	5th imputation OR (95% CI)	Pooled estimates **
Ataxia of gait	No*	1	1	1	1	1	1	1
	Yes	5.23 (2.52-10.86)	4.61 (2.43- 8.74)	5.24 (2.81-9.76)	4.55 (2.39-8.67)	5.84 (3.01-11.35)	4.36 (2.3-8.24)	4.89 (2.45-9.77)
Heel to shin	No*	1	1	1	1	1	1	1
	Yes	7.5 (3.54-15.88)	5.93 (3.02-11.64)	5.43 (2.84-10.38)	6.98 (3.5-13.91)	6.89 (3.42-13.85)	5.57 (2.86-10.84)	6.12 (2.98-12.60)
Grey to bluish discoloration of the	No*	1	1	1	1	1	1	1
	Yes	7.27 (3.01-17.57)	6.23 (2.87-13.53)	5.59 (2.66-11.77)	8.16 (3.71-17.94)	8.08 (3.63-17.96)	7.24 (3.36-15.61)	6.98 (2.98-16.38)
Dysdiadochokinesis	No*	1	1	1	1	1	1	1
	Yes	8.16 (3.8-17.53)	7.46 (3.79-14.67)	5.7 (3-10.85)	7.97 (3.99-15.92)	7.81 (3.86-15.78)	6.36 (3.28-12.36)	7.00 (3.33-14.74)
Excessive	No*	1	1	1	1	1	1	1
	Yes	19.43 (7.22-	18.95 (8.01-44.85)	16.86 (7.32-38.85)	17.08 (7.12-40.96)	23.57 (9.37-59.27)	15.86 (6.77-37.19)	18.28 (7.20-46.40)
Matchbox- test	≤17	1	1	1	1	1	1	1
	>17	8.99 (3.66-22.05)	7.40 (3.41-16.07)	6.42 (3.03-13.59)	8.31 (3.79-18.24)	8.06 (3.61-17.95)	7.09 (3.27-15.38)	7.42 (3.31-16.66)
Proteinuria	No*	1	1	1	1	1	1	1
	Yes	10.3 (3.73-28.41)	10.70 (4.32-26.53)	6.60 (2.81-15.49)	10.44 (4.35-25.08)	11.04 (4.46-27.29)	8.75 (3.71-20.64)	9.35 (3.46-25.23)
Sleep	No*	1	1	1	1	1	1	1
	Yes	4.85 (2.17-10.84)	4.81 (2.35-9.84)	5.04 (2.52-10.06)	4.13 (2.01-8.48)	4.17 (2.01-8.64)	5.03 (2.45-10.35)	4.62 (2.19-9.74)
Pencil tapping-test	≤45	1	1	1	1	1	1	1
	>45	13.9 (6.04-31.95)	8.61 (4.28-17.30)	7.17 (3.7-13.91)	9.72 (4.82-19.61)	10.14 (4.93-20.87)	10.43 (5.17-21.04)	9.13 (4.22-19.75)
Tremor at work	No*	1	1	1	1	1	1	1
	Yes	12.9 (4.9-33.96)	9.27 3.95 21.76	6.54 (2.92-14.63)	9.96 (4.18-23.72)	9.74 (4.12-23.02)	8.49 (3.67-19.63)	8.7 (3.46-21.88)
Hg in hair (µg/g)	≤1*	1	1	1	1	1	1	1
	>1 to	108.63 (19.7-598.85)	22.23 (6.65-74.34)	11.24 (3.91-32.32)	61.21 (14.04-266.83)	58.94 (17.63-197.08)	25.34 (7.38-86.98)	29.63 (4.00-219.34)
	>5	2096.27 (271.94-16159.36)	341.31 (81.88-1422.65)	140.2 (40.48-485.57)	1219.12 (219.6-6767.86)	917.84 (220.2-3825.79)	423.09 (98.65-1814.48)	468.85 (43.41-5063.98)
Hg in urine (µg/l)	≤ 7*	1	1		1	1	1	1
	>7 to	4.14 (1.69-10.15)	4.78 (2.14-10.66)	4.14 (1.92-8.92)	3.47 (1.61-7.47)	4.97 (2.23-11.09)	3.63 (1.7-7.77)	4.16 (1.77-9.77)
	> 25	78.55 (25.43-242.64)	64.00 (24.46-167.43)	59.62 (24.07-147.68)	41.89 (16.73-104.91)	64.52 (24.66-168.81)	47.15 (19.27-115.4)	54.62 (19.64-151.95)
**BIC**		**345.32**	**348.14**	**363.91**	**347.38**	**336.33**	**353.11**	**349.77 *** **

*** reference group ** calculated according to Rubin's rules *** averaged over five imputation datasets**
