# Supplementary material for: Essential Indicators Identifying Chronic Inorganic Mercury Intoxication: Pooled Analysis across Multiple Cross-Sectional Studies
Source: PLoS One. 2016 Aug 30;11(8):e0160323. doi: 10.1371/journal.pone.0160323 (PMC5004870; doi:10.1371/journal.pone.0160323)
Supplement: S3 Table — (PDF) [file pone.0160323.s003.pdf]

Additional file 3. Model 3 after stepwise variable selection in logistic regression

Indicator		Pre- imputation	Post- imputation					
		Complete case OR (95% CI)	1st imputation OR (95% CI)	2nd imputation OR (95% CI)	3rd imputation OR (95% CI)	4th imputation OR (95% CI)	5th imputation OR (95% CI)	Pooled estimates ** OR (95% CI)
Ataxia of gait (walking)	No*	1	1	1	1	1	1	1
	Yes	6.55 (3.59-11.94)	4.70 (2.75-8.04)	5.84 (3.38-10.11)	4.40 (2.64-7.33)	5.24 (3.04-9.04)	4.94 (2.93-8.33)	5.00 (2.80-8.95)
Heel to shin ataxia	No*	1	1	1	1	1	1	1
	Yes	4.02 (2.17-7.46)	3.34 (1.90-5.88)	3.84 (2.16-6.83)	3.28 (1.91-5.63)	3.64 (2.06-6.45)	3.17 (1.83-5.49)	3.45 (1.92-6.19)
Grey to bluish discoloration of the oral cavity	No*	1	1	1	1	1	1	1
	Yes	4.61 (2.27-9.35)	4.12 (2.16-7.85)	4.42 (2.31-8.47)	3.77 (2.05-6.93)	4.49 (2.33-8.65)	4.13 (2.22-7.69)	4.18 (2.18-8.03)
Dysdiadochokinesis	No*	1	1	1	1	1	1	1
	Yes	5.69 (3.06-10.58)	5.13 (2.93-8.97)	4.58 (2.62-8.02)	4.20 (2.49-7.09)	4.49 (2.57-7.86)	4.03 (2.37-6.87)	4.47 (2.5-8)
Excessive salivation	No*	1	1	1	1	1	1	1
	Yes	16.8 (7.51-37.57)	13.24 (6.50-26.99)	13.52 (6.61-27.66)	9.09 (4.70-17.56)	11.68 (5.73-23.80)	9.81 (5.02-19.17)	11.33 (5.13-25.00)
Loss of hair	No*	1	1	1	1	1	1	1
	Yes	8.25 (3.49-19.53)	5.94 (2.74-12.87)	5.53 (2.55-12.02)	4.47 (2.18-9.19)	6.33 (2.9-13.83)	4.99 (2.39-10.41)	5.41 (2.4-12.21)
Matchbox- test	≤17	1	1	1	1	1	1	1
	>17	4.10 (2.07-8.13)	4.11 (2.18-7.73)	3.81 (2.02-7.17)	3.50 (1.92-6.38)	3.98 (2.11-7.54)	3.49 (1.90-6.41)	3.77 (1.98-7.17)
Proteinuria	No*	1	1	1	1	1	1	1
	Yes	3.32 (1.49-7.40)	3.60 (1.74-7.44)	3.75 (1.80-7.81)	4.10 (2.04-8.23)	4.65 (2.22-9.75)	4.12 (2.03-8.37)	4.03 (1.90-8.55)
Sleep disturbances	No*	1	1	1	1	1	1	1
	Yes	3.76 (1.96-7.18)	2.97 (1.64-5.40)	3.08 (1.69-5.59)	2.90 (1.63-5.13)	2.87 (1.57-5.24)	3.03 (1.70-5.40)	2.97 (1.64-5.37)
Pencil tapping-test	≤45	1	1	1	1	1	1	1
	>45	4.02 (2.18-7.41)	3.72 (2.13-6.50)	3.75 (2.13-6.59)	3.63 (2.13-6.20)	3.56 (2.03-6.26)	3.85 (2.23-6.65)	3.7 (2.12-6.46)
Hg in urine (µg/l)	≤ 7*	1	1	1	1	1	1	1
	>7 to	2.21 (0.75-6.46)	2.44 (0.96-6.24)	1.51 (0.58-3.92)	2.25 (0.91-5.55)	3.01 (1.17-7.72)	2.44 (0.98-6.06)	2.28 (0.78-6.68)
	> 25	19.84 (5.45-72.19)	15.32 (4.88-48.14)	10.96 (3.45-34.83)	13.56 (4.48-41.04)	19.06 (6-60.52)	16.3 (5.31-50.07)	14.79 (4.36-50.17)
Hg urine/crea (µg/g)	≤5*	1	1		1	1	1	1
	>5 to	9.57 (3.06-29.91)	5.92 (2.21-15.83)	8.43 (3.05-23.32)	4.12 (1.61-10.54)	5.32 (1.98-14.26)	3.78 (1.47-9.73)	5.29 (1.61-17.38)
	>20	26.5 (6.8-102.73)	14.78 (4.53-48.27)	20.34 (5.97-69.38)	9.59 (3.11-29.62)	13.21 (4.04-43.26)	9.17 (2.93-28.64)	12.84 (3.27-50.47)
**BIC**		**453.26**	**452.67**	**448.13**	**491.16**	**446.32**	**477.01**	**463.06 *** **

* reference group ** calculated according to Rubin's rules *** averaged over five imputation datasets
